# Pediatric Mandibular Fractures: A Review

**DOI:** 10.5005/jp-journals-10005-1022

**Published:** 2009-08-26

**Authors:** Sunil Sharma, Abhishek Vashistha, Ankita Chugh, Dinesh Kumar, Urvashi Bihani, Mridula Trehan, Anant G Nigam

**Affiliations:** 1 Vice Principal and Head, Department of Oral and Maxillofacial Surgery, Mahatma Gandhi Dental College and Hospital Jaipur, Rajasthan, India; 2 Reader, Department of Oral and Maxillofacial Surgery, Mahatma Gandhi Dental College and Hospital, Jaipur, Rajasthan, India; 3 Senior Lecturer, Department of Oral and Maxillofacial Surgery, Mahatma Gandhi Dental College and Hospital Jaipur, Rajasthan, India; 4 Senior Lecturer, Department of Oral and Maxillofacial Surgery, Mahatma Gandhi Dental College and Hospital Jaipur, Rajasthan, India; 5 Professor, Department of Oral and Maxillofacial Surgery, Mahatma Gandhi Dental College and Hospital, Jaipur Rajasthan, India; 6 Professor and Head, Department of Orthodontics and Dentofacial Orthopedics, Mahatma Gandhi Dental College and Hospital, Jaipur, Rajasthan, India; 7 Reader, Department of Pedodontics, Mahatma Gandhi Dental College and Hospital, Jaipur, Rajasthan, India

**Keywords:** Pediatric trauma, pediatric mandibular fractures, circummandibular wiring.

## Abstract

The pattern of craniomaxillofacial fractures seen in children and adolescents varies with evolving skeletal anatomy and socioenvironmental factors. The general principles of treating mandibular fractures are the same in children and adults: Anatomic reduction is combined with stabilization adequate to maintain it until bone union has occurred. But recognition of some of the differences between children and their adult counterparts is important in long-term esthetic and functional facial rehabilitation as effect of injury, treatment provided has a great influence on their ensuing growth.

## INTRODUCTION

**Fig. 1:**
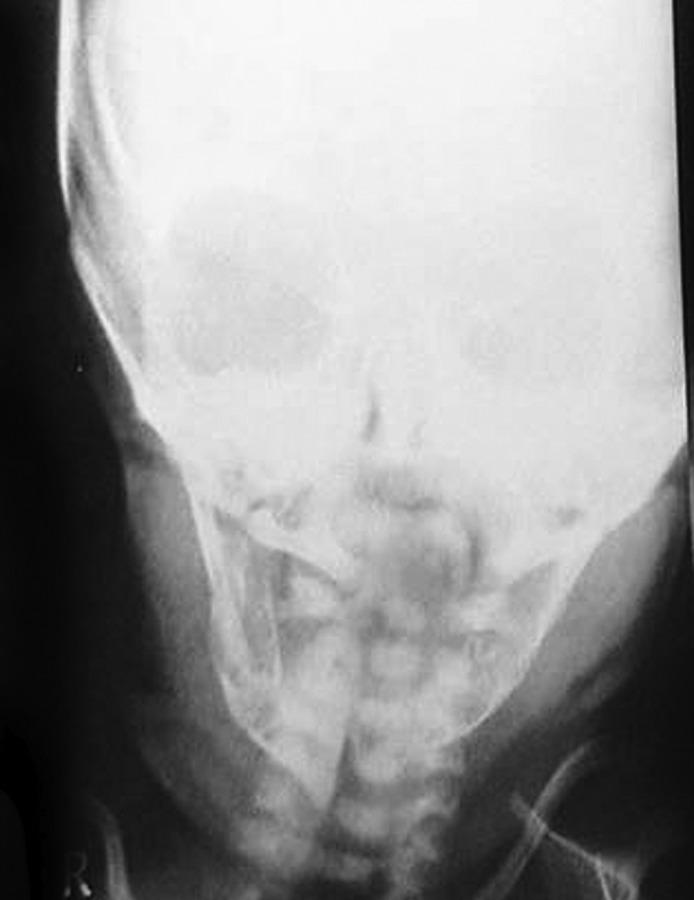
Preoperative radiograph

**Fig. 2:**
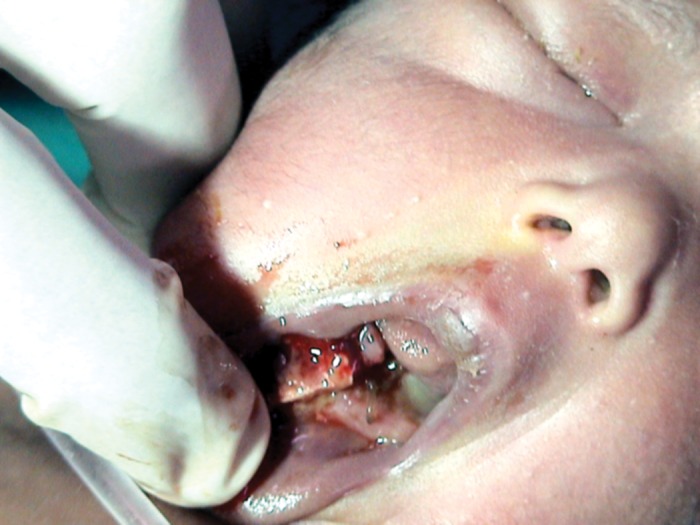
Preoperative photograph showing displaced fracture

**Fig. 3:**
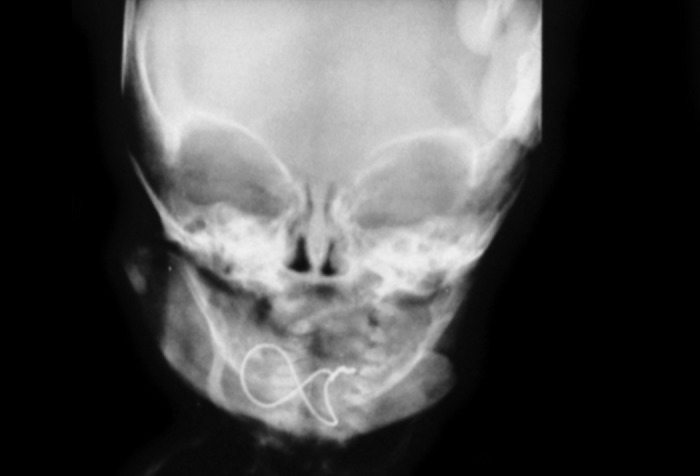
Postoperative radiograph with figure of eight wiring

In childhood a generally impetuous nature and adventurous spirit combine to encourage participation in physical activities with little thought to immediate consequences, still paradoxically facial injuries in children are much less common than adults. Above all the immense capacity for healing in children within the shortest possible time with minimum of complications, the assistance that growth can give, and the inherent ability to adapt to new situation are quite different from what we see in adults. The principles involved in treatment of facial injuries are same irrespective of the age of patient. However the techniques in children are necessarily modified by certain anatomical, physiological and psychological factors (Figs 1 to 3).

This article aims to cover comprehensively the review of these modifications and preferable options for the management of mandibular fractures in these children.

## GENERAL CONSIDERATIONS

Mandibular fractures are the most common facial skeletal injury in pediatric trauma patients.^1^-^3^ In Posnick and colleagues’ study thirty-nine percent of all fractures were of the mandible. Mandibular fracture sites included the condyle (59 of 107, 55%), parasymphysis (29 of 107, 27%), body (10 of 107, 9%) and angle (9 of 107, 8%).^4^

Young bone possesses unique physical properties that coupled with space occupying developing dentition give rise to patterns of fracture not seen in adults. Bone fragments in children may become partially united as early as 4 days and fractures become difficult to reduce by seventh day.^5^ This results in need for different forms of fixation as early as possible for comparatively shorter duration of time. Nonunion or fibrous union rarely occurs in children and excellent remodeling occurs under the influence of masticatory stresses even when there is imperfect apposition of bone surfaces. The management of mandibular fractures in children differs somewhat from that of adults mainly because of concern for possible disruption of growth. In children the final result is determined not merely by initial treatment but by the effect that growth has on form and function.

**Fig. 4:**
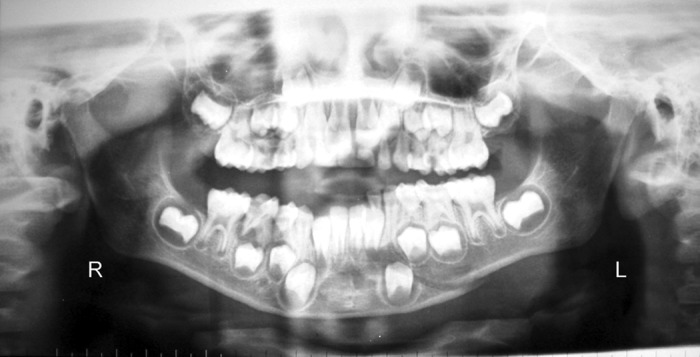
Left parasymphysis fracture

**Fig. 5:**
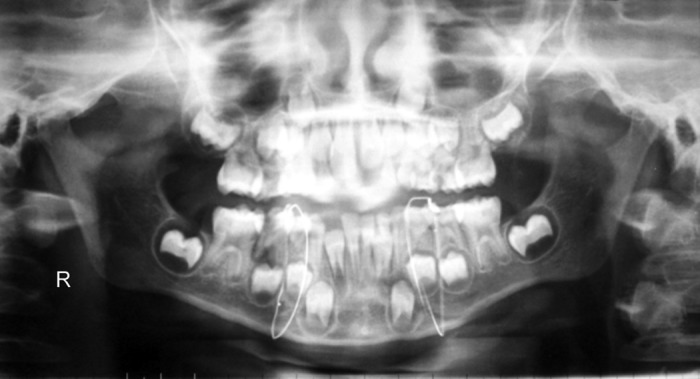
Circummandibular wiring for acrylic occlusal splint

Growth abnormalities may occur as result of fracture dislocation of condyle due to elimination of ‘functional matrix’ of lateral pterygoid function, trismus or ankylosis. Methods of dentoalveolar stabilization also require some reforms. Between 2-4 years sufficient number of fully formed deciduous teeth are present facilitating application of arch bars or eyelet wires. 5 to 8 years age old group may present with some difficulty owing to loss or loosening of deciduous teeth. The shape and shortness of deciduous crowns may make the placement of circumdental wires and arch bar slightly more difficult in children. However the narrow cervix of tooth in relation to crown and roots provides better retention of wires as in Ivy loops or stout wires. Mandibular cortex is thinner in children so care must be taken to avoid pulling a wire through the mandible when placing circummandibular wiring for splints (Figs 4 and 5).

While doing open reduction and fixation presence of tooth buds throughout the body of mandible must be a consideration as trauma to developing tooth buds may result in failure of eruption of permanent teeth and hence narrow alveolar ridge. However according to Koenig et al 82% of tooth buds in line of fracture erupted normally regardless if method of treatment was open reduction with rigid fixation or closed reduction.^6^

## EMERGENCY MANAGEMENT

The emergency management of facial trauma in pediatric population also needs extra-consideration. Clinical signs of shock may occur with even insignificant amounts of rapid blood loss due to small blood volume. Because of small size of airway laryngeal edema or retroposition of base of tongue may produce sudden obstruction. Tracheostomy if required should be done using vertical incision avoiding first tracheal ring and high lying left innominate vein.

## SPECIFIC MANAGEMENT BASED ON MANDIBULAR FRACTURE REGIONS

### Body and Symphysis

Majority of body and symphysis fractures in children are undisplaced because of elasticity of mandible and embedded tooth buds that hold the fragments together ‘like glue.’

Bilateral fractures of anterior mandible occur with much greater frequency in children than in adults. A common fracture pattern not seen in adults run from upper border beside the last tooth anteroinferiorly to the lower border in region of canine. These fractures are generally greenstick and require no active treatment.

Slight occlusal discrepancies resulting from lack of perfect reduction correct spontaneously as permanent teeth erupt and bone undergoes remodeling with function. Nondisplaced body or symphysis fractures without malocclusion can be treated by close observation, blenderized diet and avoidance of physical activity. If displaced closed reduction and immobilization is performed. Exact method of immobilization depends on child’s chronologic age and state of dental development. In under 2 years age very littl anchorage can be taken from teeth as most are unerupted or incompletely formed. In mixed dentition only 6 years molars are adequate for circumdental wires. If possible arch bars are placed and elastic immobilization is done. If teeth are inadequate then fracture site is immobilized with gunning splint or lingual splint. Intermaxillary fixation is used if splint stabilization is not enough as in fracture of posterior body beyond point of extension of splint. Appliance should be fixed in place using circummandibular wires one on either side of fracture and two wires to add stability to the splint. If IMF is also required then wires can be added from circummandibular wires to wires at piriform region or zygoma. Splint should be left in place for three weeks. Alternatively if possible monocortical plate at inferior border can be placed. Short (4 mm) and broader screws 2 mm should be used as they are more retentive in pediatric bone.

The common occurrence of a combined parasymphyseal and condylar fracture will warrant a more stable form of parasymphyseal fracture fixation (miniplates and screws) so that early active mandibular range of motion with TMJ function can occur.

## Angle

Fractures at angle proximal to tooth bearing area are not sufficiently immobilized with splint alone so closed reduction and intermaxillary fixation for 3 weeks are required. When tightening splints sawing of wire through the mandible has to be avoided.

When a mandibular angle fracture occurs in the presence of a condyle fracture, the combined forces may be significant enough to cause displacement unless ORIF at the angle fracture is carried out. Plating at the tension-band zone is not recommended in the mixed dentition. In open reduction for less than 5 years it is possible to injure tooth buds near angle when placing intraosseous wire or screws which requires caution.

## Condyle

Trauma to chin producing temporomandibular joint injury is frequent occurrence in childhood. Mandibular condyle in children is short, stout and highly vascular with thin cortical plate. The impact displaces condyle posterosuperiorly against skull base thus leading to range of injury from capsular tear, hemarthrosis to fracture of condylar head or neck. Occasionally a crush injury to condyle can produce comminuted fracture. Children less than 3 years of age with trauma to condyle are at greatest potential for growth disturbance especially due to ankylosis. Inadequate or overtreatment may lead to growth retardation or excess while excessive immobilization may lead to mandibular hypomobility.^7^ So the two main goals for treatment in such patients are (1) Preservation of function (2) Maintenance of ramus height. When this is achieved normal growth usually occurs.

Unlike adults a child with fracture condyle frequently presents with midline deviation away from rather than toward the injury owing to swelling or hematoma within the joint. The location and degree of displacement of condylar fractures in children in primary and mixed dentition stage is not that useful variable for developing treatment plan.^1^,^2^ Rather the amount of interincisal opening dental age, occlusion and level of pain must be assessed carefully. If these are normal close observation and blenderized diet can be the treatment option. Nonoperative management (observation, exercises, maxillomandibular fixation, training elastics, bite opening splints) are overwhelmingly popular because there are minimal complications and outcomes are good with adults and children alike. Open reduction with internal fixation is rarely indicated for pediatric condylar or subcondylar fractures.

In intracapsular injuries especially in less than 3 year of age as chances of ankylosis are high mandibular exercises and jaw stretching should be started early to avoid such complications.^8^ For older children muscle relaxants, jaw stretching exercises help to achieve normal function.

In children in primary and mixed dentition stage with unilateral condylar fractures analgesics and blenderized diet for 5-7 days is usually adequate treatment. Minor malocclusions will correct spontaneously. Deviation on opening is treated with midline opening exercises.^9^ If there is significant pain and severe malocclusion short period of immobilization for 7-10 days with or without bite opening splint is indicated. This can be followed with training elastics. In bilateral subcondylar fractures in children in primary and mixed dentition stage relatively normal opening and stable occlusion may be present. Analgesics and blenderized diet for 7-10 days followed by soft diet for two weeks may be adequate. However bilateral fractures with significant dislocation often produce open bite malocclusion. In these cases jaw should be immobilized for 7-10 days and after release of fixation guiding elastics for 7-10 days should be given, if still malocclusion persists then open reduction should be considered.

In permanent dentition stage with unilateral or bilateral condylar fractures especially if dislocation present with persistent malocclusion after 7-10 days of intermaxillary fixation open reduction to restore ramus length and to prevent progressive deformity must be considered as in older children there is less capacity for bone to adapt and remodel.

Restoration of normal symmetric jaw function provides best chance for normal growth.

Open reduction of a condyle fracture may be warranted in a child in some instances. Indications may include the following:

- Displacement into the middle cranial fossa.
- Unacceptable occlusion after a closed technique trial has failed or mechanical obstruction present.
- Avulsion of the condyle from the capsule.
- Bilateral fractures of the condyles with comminuted midface fractures.
- Penetrating wound is present.

In all other cases conservative nonoperative management produces equally acceptable result with minimal complication.

## MANDIBULAR FRACTURES AND GROWTH ABNORMALITIES

Decreased vertical height of mandibular body and alveolus may occur after fracture of horizontal ramus of mandible if teeth are lost due to injury or hardware through tooth buds. Contour defects may occur due to severely comminuted or compound fractures when bone undergoes resorption during remodeling. In general however mandibular body fractures present little risk for long-term growth abnormalities. Unilateral and bilateral condylar fractures may however cause mandibular asymmetry and retrognathism with open bite respectively. Leake et al^9^ demonstrated no growth abnormalities in 13 children with unilateral and 8 children with bilateral subcondylar fractures treated with analgesics, liquid diet, exercises and guiding elastics. According to Kaban 1 out of 39 patients developed slight asymmetry after subcondylar fracture. Mac lenan found late facial growth deformities in patients with intracapsular fractures prior to age 2.5 years.

Lund 6 carried out a prospective study of 38 patients with subcondylar fractures to study the effect of injury on mandibular growth and the extent of remodeling that took place. Of 38 patients 32 were 12 years or less and 6 were 13-17 years. There were 11 bilateral fractures and 27 unilateral fractures. 35 patients were treated with close observation alone or in combination with intermaxillary fixation. Three patients had open reduction and fixation with K-Rod (1) and condylectomy (2). In Lund’s study mandibular growth was generally greater on fractured side than nonfractured side so that the fractured ramus which was initially shorter had greater incremental growth rate so that possible disproportion between two sides reduced with time. This was evident when measuring distance between chin point to condyle.

Three types of mandibular growth patterns were noted:

1. Compensatory growth without overgrowth. Fractured side grows more but in end remain somewhat shorter than normal (13 of 27 patients).
2. Compensatory growth with overgrowth. Fractured site grows longer than normal (8 of 27 patients).
3. Dysplastic growth. Fractured site grows less so difference is accentuated with time.

Compensatory growth mainly occurred in patients growing at time of injury.

Lund also defined two groups on basis of pattern of remodeling.

1. Incomplete remodeling in which condyle was irregular or displacement remained at fracture site (12 of 49 condyles).
2. Complete remodeling (37 out of 49 condyles).

He concluded that patients with displaced condyle had greater chance of incomplete remodeling. Successive radiographs showed that remodeling consisted of resorption and apposition. The process began at time of injury and continued for period of 5 to 49 months. When remodeling occurred completely it consisted of resorption of proximal condylar stump and outgrowth of bony process on ramus resembling normal condyle.

## NEWER TRENDS

Earlier most of the pediatric cases were treated with conservative measures or closed reduction techniques. Only recently have the distinct advantages of accurate primary repair and the stable fixation of facial fractures been applied to the rehabilitation of injuries in children too.^10^ With the advent of better investigative facilities like CT scan and 3D reconstruction, and newer airway management techniques with reliable anesthesia techniques and specifically introduction of mini and microplates open reduction and fixation of pediatric facial fractures is getting commoner.

Also, resorbable materials have been made available as a fixation option for pediatric craniomaxillofacial fracture management. According to Peterson with the exception of mandibular condyle fractures judicious use of ORIF is preferable to the closed reduction and immobilization techniques with splints when treating fractures in the deciduous and mixed dentition.^11^

## CONCLUSION

Mandibular fractures in children most commonly occur in condylar region, followed by parasymphysis and angle. The fractures tend to be minimally displaced and in majority of cases can be treated conservatively. Significantly displaced mandibular fractures are reduced and immobilized using rigid internal fixation according to principles used in adults. Fractures in condylar region usually are treated using nonoperative therapies as in most cases fracture heals and condyle is remodeled with successful anatomic and functional results.
